# Different Aspects of Classical Pathway Overactivation in Patients With C3 Glomerulopathy and Immune Complex-Mediated Membranoproliferative Glomerulonephritis

**DOI:** 10.3389/fimmu.2021.715704

**Published:** 2021-08-11

**Authors:** Marloes A. H. M. Michels, Nicole C. A. J. van de Kar, Sanne A. W. van Kraaij, Sebastian A. Sarlea, Valentina Gracchi, Flore A. P. T. Engels, Eiske M. Dorresteijn, Johannes van der Deure, Caroline Duineveld, Jack F. M. Wetzels, Lambertus P. W. J. van den Heuvel, Elena B. Volokhina

**Affiliations:** ^1^Department of Pediatric Nephrology, Radboud Institute for Molecular Life Sciences, Amalia Children’s Hospital, Radboud University Medical Center, Nijmegen, Netherlands; ^2^Department of Laboratory Medicine, Radboud University Medical Center, Nijmegen, Netherlands; ^3^Department of Pediatric Nephrology, Beatrix Children’s Hospital, University Medical Center Groningen, University of Groningen, Groningen, Netherlands; ^4^Department of Pediatric Nephrology, Maastricht University Medical Center+, Maastricht, Netherlands; ^5^Department of Pediatric Nephrology, Sophia Children’s Hospital, Erasmus Medical Center, Rotterdam, Netherlands; ^6^Department of Pediatrics, Deventer Hospital, Deventer, Netherlands; ^7^Department of Nephrology, Radboud Institute for Health Sciences, Radboud University Medical Center, Nijmegen, Netherlands; ^8^Department of Pediatrics/Pediatric Nephrology, University Hospitals Leuven, Leuven, Belgium; ^9^Department of Development and Regeneration, University Hospitals Leuven, Leuven, Belgium

**Keywords:** C3 glomerulopathy, classical complement pathway, autoantibody, C3 convertase, C4 nephritic factors (C4NeFs), C3 nephritic factors (C3NeFs)

## Abstract

The rare and heterogeneous kidney disorder C3 glomerulopathy (C3G) is characterized by dysregulation of the alternative pathway (AP) of the complement system. C3G is often associated with autoantibodies stabilizing the AP C3 convertase named C3 nephritic factors (C3NeF). The role of classical pathway (CP) convertase stabilization in C3G and related diseases such as immune complex-mediated membranoproliferative glomerulonephritis (IC-MPGN) remains largely unknown. Here, we investigated the CP convertase activity in patients with C3G and IC-MPGN. Using a refined two-step hemolytic assay, we measured the stability of CP convertases directly in the serum of 52 patients and 17 healthy controls. In four patients, CP convertase activity was prolonged compared to healthy controls, i.e. the enzymatic complex was stabilized. In three patients (2 C3G, 1 IC-MPGN) the convertase stabilization was caused by immunoglobulins, indicating the presence of autoantibodies named C4 nephritic factors (C4NeFs). Importantly, the assay also enabled detection of non-immunoglobulin-mediated stabilization of the CP convertase in one patient with C3G. Prolonged CP convertase activity coincided with C3NeF activity in all patients and for up to 70 months of observation. Crucially, experiments with C3-depleted serum showed that C4NeFs stabilized the CP C3 convertase (C4bC2a), that does not contain C3NeF epitopes. All patients with prolonged CP convertase activity showed clear signs of complement activation, i.e. lowered C3 and C5 levels and elevated levels of C3d, C3bc, C3bBbP, and C5b-9. In conclusion, this work provides new insights into the diverse aspects and (non-)immunoglobulin nature of factors causing CP convertase overactivity in C3G/IC-MPGN.

## Introduction

C3 glomerulopathy (C3G) is an umbrella term for kidney diseases characterized by glomerular inflammation and accumulation of breakdown fragments of complement component C3 in the glomeruli (1, 2). The diagnosis requires a kidney biopsy showing C3 staining that is dominant over other immune reactants by immunofluorescence microscopy. The two major subgroups within the classification of C3G are dense deposit disease (DDD) and C3 glomerulonephritis (C3GN). DDD is distinguished from C3GN by characteristic, highly electron-dense, intramembranous sausage-shaped deposits in electron microscopy (1, 2). C3G patients can present with various symptoms, such as proteinuria, hematuria, hypertension, and decreased kidney function. Current therapy encompasses antihypertensive agents and immunosuppressive drugs such as prednisone and mycophenolate mofetil. Despite this treatment, many patients progress to kidney failure within ten years (2, 3).

Before the definition of C3G was introduced in 2010 (4) and consensus on the definition was further implemented by a group of specialists in 2012-2013 (1), the membranoproliferative glomerulonephritis (MPGN) classification was used by physicians. This is a rather non-specific pathology term that besides C3G includes immune complex-mediated MPGN (IC-MPGN), which is characterized by significant staining for immunoglobulins (Igs). The new umbrella classification of C3G was developed to emphasize the underlying disease process: the dysregulation of the alternative pathway (AP) of the complement system. This was meant to encourage the pathologist and nephrologist to start investigations of complement abnormalities, also in view of complement-targeting drugs that are being developed (1, 4). Indeed, in 40-90% of all C3G cases Igs binding the central AP enzyme, the AP C3 convertase [C3bBb(P)], are found in the blood (5–8). These autoantibodies are named C3 nephritic factors (C3NeFs) and stabilize the convertase complex, prolonging its C3-cleaving activity. In addition, aberrations in genes encoding AP components and/or regulators can be found in approximately 20% of C3G patients (5, 8). Nonetheless, a recent whole-genome sequencing study found that not rare complement gene variations, but HLA type was associated with C3G, implicating that an autoimmune mechanism underlies the disease in most cases (9). Interestingly, abovementioned AP dysregulations such as C3NeFs are also found in a substantial number of IC-MPGN patients (5, 10, 11). In some cases, distinguishing between C3G and IC-MPGN based on immunofluorescence microscopy remains difficult, and some patients with IC-MPGN may evolve to C3G and vice versa (2). In a small number of patients still no pathogenic acquired or genetic factors can be identified.

The AP serves as a surveillance mechanism for complement activity due to its constant low rate of activation. In addition, the pathway encompasses a powerful loop to rapidly amplify the activity of all complement pathways, including the classical pathway (CP) and lectin pathway that are activated by specific pattern recognition (12–14). Although AP dysregulation has been extensively studied in the context of C3G, less research has focused on the involvement of CP convertase dysregulation. Recently, C4 nephritic factors (C4NeFs) have been reported in three C3G patient cohorts with a prevalence of 3-15% (15–17). In analogy with C3NeFs, C4NeFs are autoantibodies that bind and stabilize the CP C3/C5 convertases [C4bC2a(C3b)] resulting in a prolongation of the half-life of these enzyme complexes from minutes to hours. C4NeFs have also been previously reported in patients with (IC-)MPGN (16, 18–20), post-infectious glomerulonephritis (21), systemic lupus erythematosus (18, 22, 23), Sjögren syndrome (18), and in one patient with sepsis caused by a *Neisseria meningitidis* infection (24). Nonetheless, studies on the presence and relevance of C4NeFs in complement-mediated kidney diseases such as C3G and IC-MPGN are still limited and often not part of the standard laboratory workup. Furthermore, there is no standardization of the different assays that are being used.

In this work, we investigated whether CP convertase overactivity occurred in our patient cohort consisting of 45 patients with C3G and 7 patients with IC-MPGN. First, we validated a two-step CP convertase activity assay measuring the stability of CP convertases directly in the serum of patients, to screen for factors prolonging the CP convertase activity. Next, we characterized the nature of these factors and investigated their prevalence in the disease course and relation to complement activation markers.

## Materials and Methods

### Sample Collection and Inclusion

**Figure 1:**
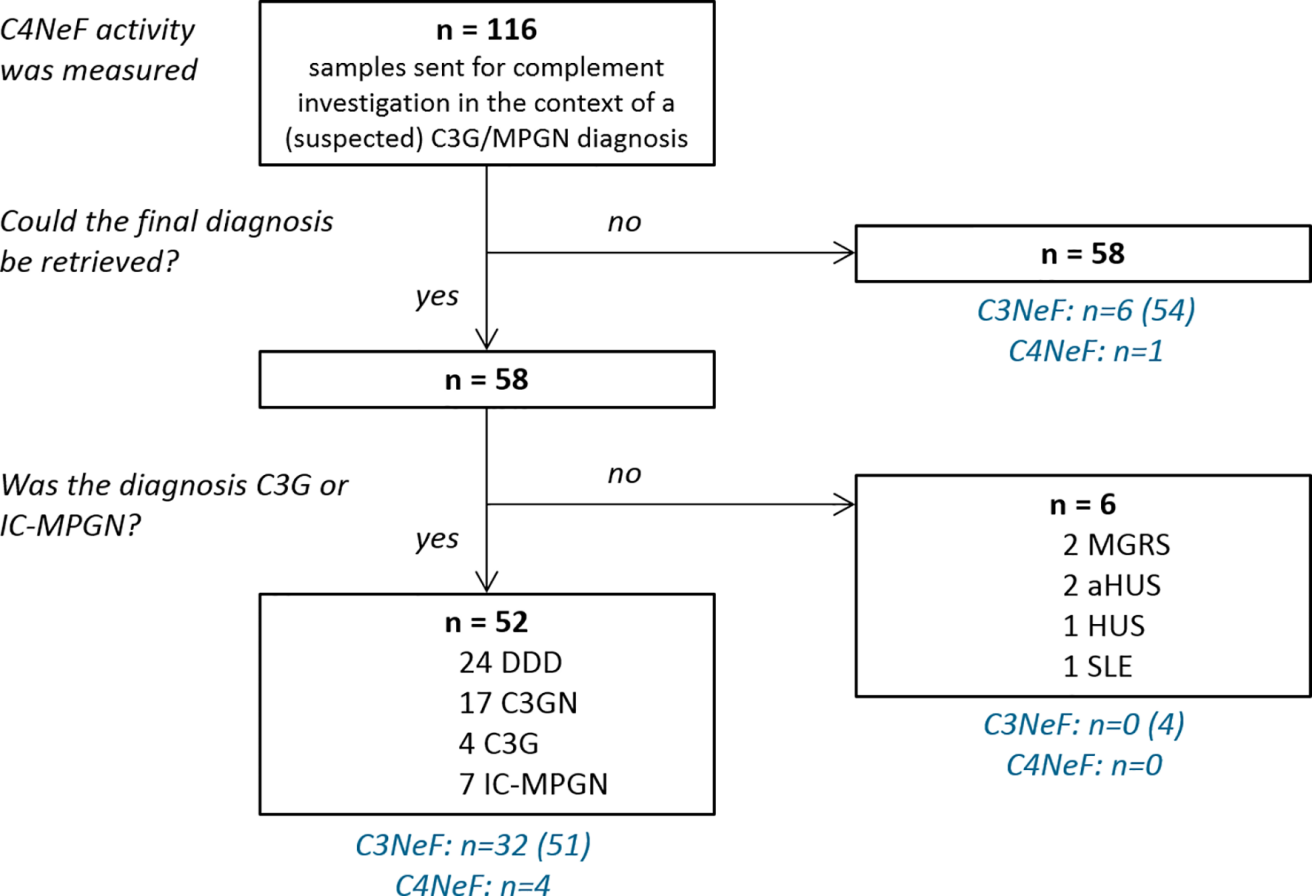
Flow chart of sample inclusion. All patient samples were measured for prolonged classical pathway activity, i.e. C4 nephritic factor (C4NeF) activity, but only the 52 patients with a final diagnosis of C3 glomerulopathy (C3G) or immune complex-mediated membranoproliferative glomerulonephritis (IC-MPGN) were included in this study. C3 nephritic factor (C3NeF) activity, i.e. prolonged alternative pathway activity, was measured in the number of patients indicated between brackets. DDD, dense deposit disease; C3GN, C3 glomerulonephritis; MGRS, monoclonal gammopathy of renal significance; HUS, hemolytic uremic syndrome; aHUS, atypical HUS; SLE, systemic lupus erythematosus.

Between July 2001 and March 2020, blood samples from 116 patients whose physicians indicated a suspicion of C3G or MPGN were sent to the Radboud university medical center for complement investigation corresponding to their suspected disease. All samples were measured for abnormal CP convertase activity. For 58 patients the final diagnosis could be retrieved (**Figure 1**). Fifty-two patients met the inclusion criteria of a final diagnosis of C3G (1) or idiopathic IC-MPGN: 45 patients had C3G (24 DDD, 17 C3GN, and 4 cases that could not be further classified due to lack of electron microscopy data) and 7 had IC-MPGN. Of note, P1 and P2 have been described before as P25 (25) and P3 (26) and P10 (26), respectively. Six patients had another glomerular diagnosis: 2 monoclonal gammopathy of renal significance, 2 atypical hemolytic uremic syndrome, 1 hemolytic uremic syndrome, and 1 systemic lupus erythematosus. These six patients and the patients with unknown definite diagnosis were excluded. Furthermore, samples collected from 17 healthy adult controls were included in this study.

The study was performed in accordance with the appropriate version of the Declaration of Helsinki. Whole blood samples were processed within 1 hour after sampling according to the standard protocol (25). To obtain normal human serum (NHS) and normal human plasma (NHP), 17 control sera and 17 control ethylenediaminetetraacetic acid (EDTA)-plasma samples were pooled, respectively.

### Sensitized Sheep Erythrocytes

Sheep erythrocytes (ShE) suspended in Alsever’s solution were obtained from Håtunalab AB (Bro, Sweden). For each experiment, freshly sensitized ShE working suspensions were prepared as described previously (27), by washing the erythrocytes in dextrose gelatin veronal buffer (DGVB++; 45 mM veronal buffer, pH 7.4, 72 mM NaCl, 139 mM D-glucose, 0.1% gelatin, 1 mM MgCl2, 0.15 mM CaCl2) and incubating them with 1000x diluted amboceptor (Siemens Healthcare, Erlangen, Germany) for 20 min at 37°C. In each experiment, erythrocyte working suspensions were calibrated to yield an absorbance of 1.8-2.2 at 405 nm when 10x diluted in water.

### Immunoglobulin Purification

The Ig fractions were isolated from 0.2-1.0 mL EDTA-plasma (P1, P4, and NHP) or serum (P2, P3, and NHS) samples as previously described (25), using Nab™ protein A/G spin columns (Thermo Fisher Scientific, Waltham, MA, USA). After purification and dialysis, Ig fractions were concentrated in phosphate buffered saline to the initial sample volume. Final protein concentrations were measured using NanoDrop Spectrophotometer (Thermo Fisher Scientific, Waltham, MA, USA) and varied between 2.4 and 9.8 mg/mL for the patient samples and between 10.1 and 11.1 mg/mL for the control pools.

### Classical Pathway Convertase Activity Assay

Activity of CP C3/C5 convertases was measured using a two-step convertase activity assay that follows the same principles as described previously (15, 28, 29) (**Supplementary Figure 1**). In the first step, test serum was incubated for different time periods with sensitized ShE in presence of a C5-inhibitor to allow CP activation and CP convertase assembly up to the level of C5 convertases. After a washing step, preformed convertase complexes were allowed to generate C5b-9 and subsequent hemolysis using guinea pig serum in presence of EDTA.

Per experimental time point, 10 µL of sensitized ShE were mixed with 20 µL DGVB++ containing 10 nM of the C5-inhibitor eculizumab (Alexion Pharmaceuticals, Cheshire, CT, USA). Subsequently, 20 µL of 1.9-2.5% test serum or EDTA-plasma diluted in DGVB++ (for a final sample concentration in the well of 0.75-1%) was added at different time points to allow convertase assembly for 20, 10, 5, 2.5, 1.0, and 0.5 min. Where applicable, NHS or C3-depleted serum (A314, Complement Technology, Tyler, TX, USA) were first mixed with purified Igs in a volume ratio of 1:1, 1:3, or 1:5 before being added as the test sample. Then, 150 µL of ice-cold 40 mM EDTA–gelatin veronal buffer (EDTA–GVB, 4.41 mM veronal buffer, 0.1% gelatin, 130 mM NaCl, pH 7.4) was added, after which the cells were washed and collected by centrifugation (2 min, 1,000 g, room temperature). For the second step, erythrocytes were incubated for 60 min with 50 µl of 2.5% guinea pig serum (Envigo, Venray, the Netherlands) in EDTA-GVB as a source of C5b-9 components, supplemented with 50 µl of DGVB++ buffer. All incubation steps above were performed in V-shaped 96-well plates (Greiner Bio-One) at 37°C with 600 rpm agitation in a VWR^®^ Incubating Microplate Shaker with 3 mm orbit (VWR International). Finally, the supernatant was collected by centrifugation and transferred to flat-bottom 96-well plates (Greiner Bio-One). Hemolysis was quantified as percentage of full lysis generated by an equal amount of erythrocytes in water: (*A*_405_ test sample − *A*_405_ blank)/(*A*_405_ full lysis − *A*_405_ blank) × 100%.

### Other Complement Investigations

C3NeF activity was measured using a two-step hemolytic AP convertase activity assay (25). Levels of C3bBbP, C3bc, C5b-9, C5, properdin, Factor H, and Factor I were measured in the appropriate EDTA-plasma or serum samples with enzyme-linked immunosorbent assays (ELISA). Protocols were described before (26, 27, 30, 31), but for the measurement of properdin in EDTA-plasma samples a monoclonal mouse anti-human properdin antibody (A233, Quidel, San Diego, CA, USA) and goat anti-human properdin antibody (Nordic-MUbio, Susteren, the Netherlands) were used. For all ELISAs patient material that gave a value outside the reference range was used as a positive control. The C3, C4, and C3d levels, and the results of the genetic analysis were obtained from the patient files. Coding regions of the complement genes *CFH*, *CFHR5*, *CFI*, *CD46*, *C3*, and *CFB* were screened for rare genetic variants (i.e. <1% allele frequency) according to previously described protocol (32). In addition, multiplex ligation-dependent probe amplification, using the SALSA^®^ P236 probe mix and reagent kits of MRC Holland (Amsterdam, the Netherlands), was performed to analyze the *CFH*/*CFHR* region in all patients.

### Statistical Analysis

Where indicated, data were analyzed using two-way analysis of variance with Bonferroni’s post-test using GraphPad Prism 5.03 for Windows (GraphPad Software, San Diego, CA, USA).

## Results

### CP Convertase Activity in Healthy Controls

**Figure 2:**
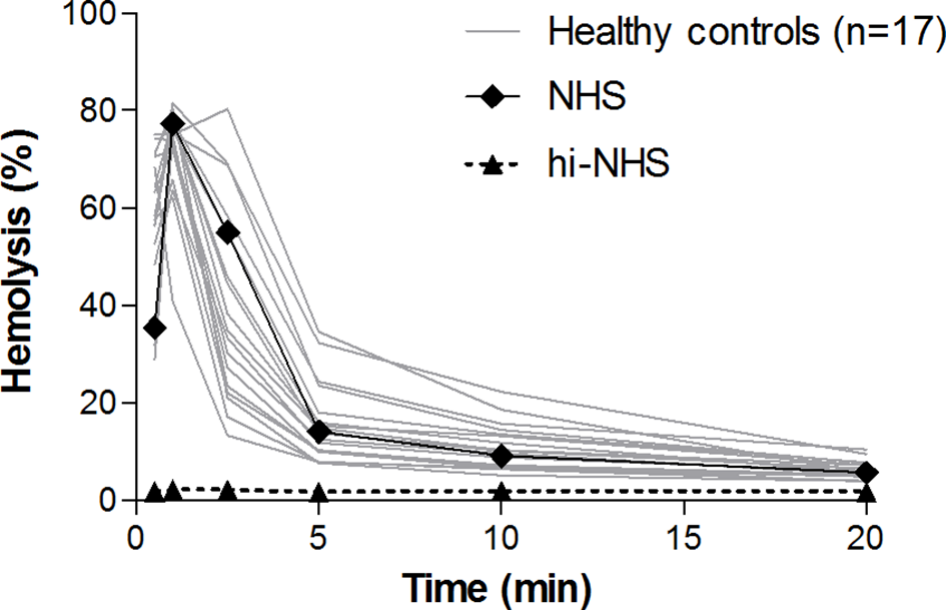
Classical pathway convertase activity in healthy controls. Classical pathway convertase activity was measured by incubating sensitized sheep erythrocytes with sera obtained from 17 healthy individuals. Normal human serum (NHS) and heat-inactivated NHS (hi-NHS) were used as assay controls. Hemolysis levels are presented as percentage of full lysis of erythrocytes in water. Results represent means of at least three independent measurements for each sample. Standard deviations have been omitted for better visibility.

To establish the normal CP convertase activity profile, 17 healthy control sera were analyzed. These individual controls showed activity profiles that were comparable to each other and to NHS (**Figure 2**). Maximal CP convertase activity was reached after 0.5-2.5 min, followed by a sharp decrease in hemolysis in all samples at 5 min of incubation and onwards.

We used these data to define cut-off criteria for detecting aberrant, prolonged convertase activity in patient samples. Analogous to the AP convertase activity assay published previously (25), we determined the ratio between the highest achieved hemolysis and the hemolysis at 10 min of incubation (top/t10 ratio) to describe the CP convertase decay. Healthy controls showed a top/t10 ratio of 7.7 ± 3.1 (mean ± SD). Prolonged activity, i.e. delayed decay, was defined as a top/t10 ratio below the mean - 2SD of healthy controls, which is below 1.6. Hemolytic activity overall was also taken into account: the area under the curve of a sample should be comparable to or higher than that of NHS to be considered as aberrant.

### CP Convertase Activity Is Prolonged in Four Patients

**Figure 3:**
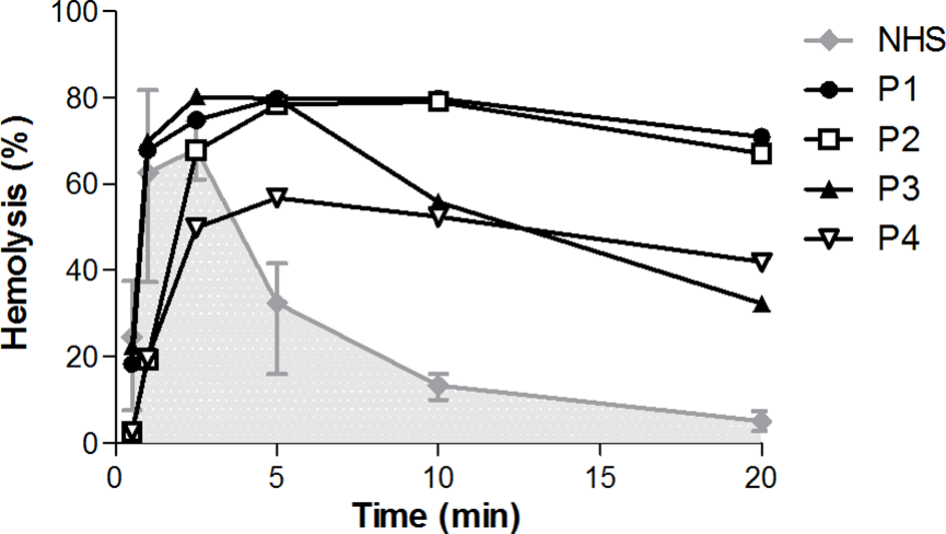
Prolonged classical pathway convertase activity profiles in four patients. Classical pathway convertase activity was measured by incubating sensitized sheep erythrocytes with normal human serum (NHS) or with patient samples. Depicted are the activity profiles for patients P1, P2, P3, and P4, that tested positive for prolonged convertase activity as their top/t10 ratios were <1.6 in at least three measurements performed. Representative runs of these samples are shown. Data for NHS are given as the mean and range from the four experiments in which the positive patients were detected. The area under the NHS curve is indicated in grey. Hemolysis levels are presented as percentage of full lysis of erythrocytes in water.

We then screened for deviant CP convertase activity in the samples of 52 C3G/IC-MPGN patients whose samples were collected for complement investigation in the context of their disease. The patients had a median (IQR) age at the time of investigation of 15 (8–18) years and 63% was female. The samples of four patients showed prolonged CP convertase activity (**Figure 3**). These activity profiles were characterized by top/t10 ratios below 1.6 and an area under the curve higher than that of NHS (**Table 1**). Three of the four patients with prolonged CP convertase activity had a biopsy-proven diagnosis of C3G (P1, P2, and P3), and one patient had biopsy-proven IC-MPGN (P4). In two patients, rare genetic variants were found in *C3* (P2) and *CFHR5* (P4) complement genes (**Table 1**). Of note, no prolonged CP convertase activity was observed in the six excluded patients diagnosed with other glomerular disorders (**Figure 1**).

**Table 1:** Details of the patients showing prolonged classical pathway convertase activity.

Patient	Gender (M/F)	Age^a^ (years)	Diagnosis	Top/t_10_ ratio [mean (range) ^b^]	AUC *vs* NHS [mean^b^ (%)]	Genetic aberrations (heterozygous)	Minor allele frequency^c^
P1	M	15	DDD	1.0 (1.0 – 1.1)	435%	–	
P2	F	16	C3GN	1.1 (1.0 – 1.2)	313%	*C3* c.691A>C (p.Ser231Arg)	Not available
P3	F	10	C3GN	1.4 (1.4 – 1.5)	218%	–	
P4	M	16	IC- MPGN	1.1 (1.1 – 1.3)	168%	*CFHR5* c.485_486dup (p.Glu163Lysfs*10)^d^	0.68%

a Age at time of sample collection for investigation.

b Obtained from three independent experiments.

c According to the gnomAD database.

d This patient additionally carries the CFH Tyr402His variant.

AUC, area under the curve; NHS, normal human serum; DDD, dense deposit disease; C3GN, C3 glomerulonephritis; IC-MPGN, immune complex-mediated membranoproliferative glomerulonephritis.

### CP Convertases Are Stabilized by Immunoglobulins (C4NeFs) in Only Three of the Four Patients

**Figure 4:**
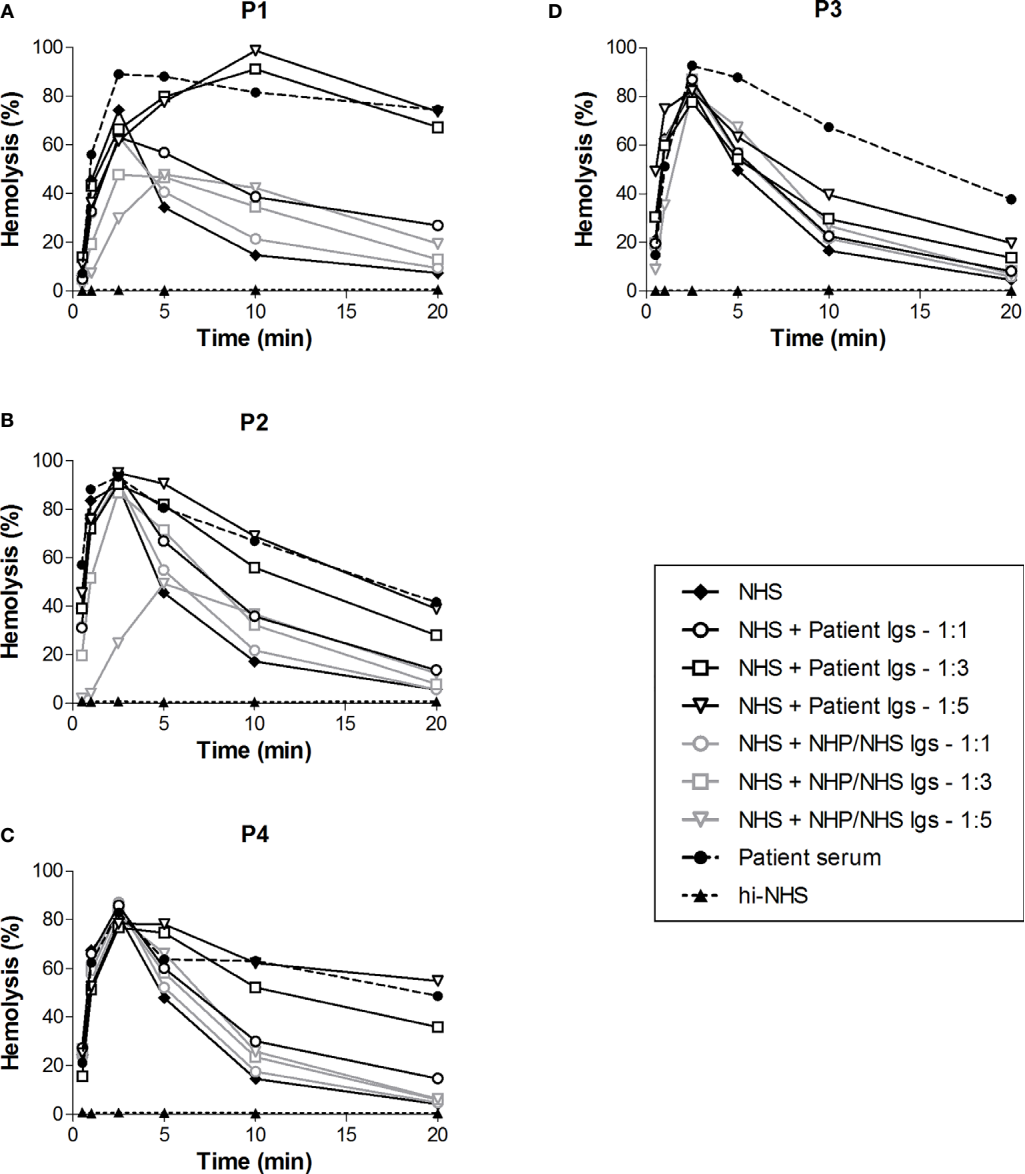
Classical pathway convertase activity profiles of control serum supplemented with patient immunoglobulin fractions. Sensitized sheep erythrocytes were incubated with normal human serum (NHS) mixed with an equal (1:1) or excess volume (1:3 and 1:5) of immunoglobulins (Igs) purified from the EDTA-plasma of patient P1 **(A)** and P4 **(C)** or from the serum of P2 **(B)** and P3 **(D)**. As a negative control, Igs purified from normal human plasma (NHP) or NHS, depending on the source of the patient Igs, were added in similar ratios. The patient sera were used as positive controls. Hemolysis levels are presented as percentage of full lysis of erythrocytes in water. Concentration series were tested once. hi-NHS, heat-inactivated NHS.

To investigate whether Igs, i.e. C4NeFs, were responsible for the increased convertase stability in the sera of patients P1-P4, we purified the Igs from the samples. Purified Ig fractions were concentrated in phosphate buffered saline to the initial sample volume and added to NHS in different volume ratios in the convertase activity assay (**Figure 4**). The Igs of P1, P2, and P4 dose-dependently prolonged the CP convertase activity, indicating C4NeF presence (**Figures 4A–C**). In contrast, the Igs of P3 did not support CP convertase stabilization, even when added in a five-fold excess to NHS (**Figure 4D**).

### C4NeF Immunoglobulins Stabilize the C4bC2a Convertase

**Figure 5:**
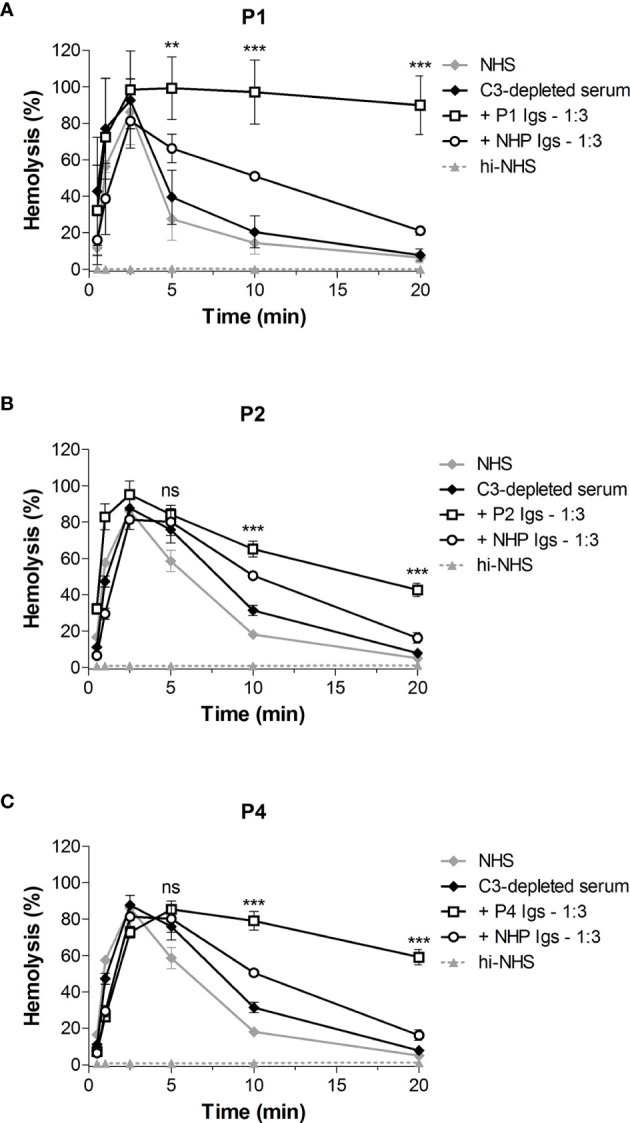
Classical pathway C3 convertase (C4b2a) stabilization by patient immunoglobulins. Sensitized sheep erythrocytes were incubated with C3-depleted serum supplemented with immunoglobulins (Igs) from P1 **(A)**, P2 **(B)**, or P4 **(C)** in a 1:3 volume ratio. Igs from normal human plasma (NHP) were used as a control. Hemolysis levels are presented as percentage of full lysis of erythrocytes in water. Data are presented as the mean and the standard deviation of three independent experiments. Significance values according to two-way analysis of variance with Bonferroni post-test with C3-depleted serum + NHP Igs as the control condition are given for t5, t10, and t20: ***P < 0.001; **P < 0.01; ns, not significant. NHS, normal human serum; hi-NHS, heat-inactivated NHS.

In the first step of the CP convertase activity assay both C3 convertases (C4bC2a) and C5 convertases (C4bC2aC3b) are assembled and further complement activation is blocked by eculizumab. C3b is also a component of AP C3 (C3bBbP) and C5 (C3bBbPC3b) convertases. Thus, to specifically analyze the CP C3 convertase-stabilizing activity of C4NeFs, C3-depleted serum was used in the first step of the assay so that only C4b2a could be formed. Addition of the Ig fractions from the patients showing Ig-mediated convertase stabilization in NHS, i.e. P1, P2, and P4, to C3-depleted serum resulted in prolonged CP C3 convertase activity profiles in all cases (**Figures 5A–C**). The hemolysis levels at t10 and t20 were significantly elevated compared to C3-depleted serum to which control Igs were added (P<0.001).

### C4NeF and C3NeF Activity During Patient Follow-Up

In the C3G/IC-MPGN cohort, 63% (29/44 C3G, 3/7 IC-MPGN) of the patients displayed prolonged AP convertase activity, i.e. C3NeF activity (**Figure 1**). All four patients that showed prolonged CP convertase activity, from now on defined as C4NeF activity regardless of the (non-)Ig nature, also displayed C3NeF activity. The C3NeF activity was Ig-mediated in all these patients. Autoantibodies to Factor H were not detected in any of the patients.

**Figure 6:**
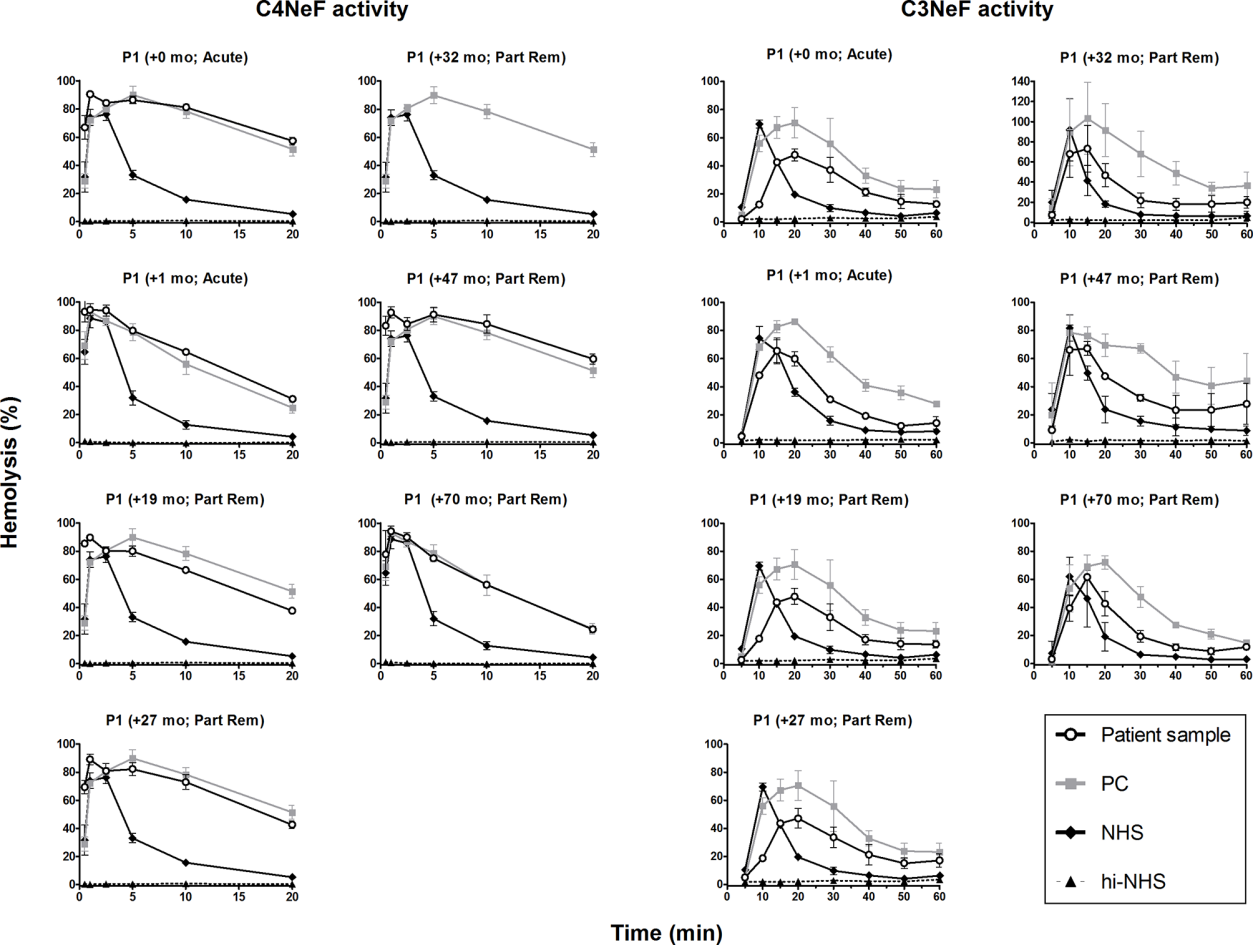
C4 nephritic factor and C3 nephritic factor activity of patient P1 during follow-up. Samples from P1 collected over the disease course were tested for prolonged classical pathway activity, i.e. C4 nephritic factor (C4NeF) activity, and for prolonged alternative pathway activity, i.e. C3 nephritic factor (C3NeF) activity. Collection dates are presented as time after acute presentation in months (+mo). Hemolysis levels are presented as percentage of full lysis of erythrocytes in water. Results are shown as mean and range of two independent experiments. For the C4NeF activity assay, the positive control (PC) is the sample of P1 collected 32 mo after presentation. Part Rem, partial remission; NHS, normal human serum; hi-NHS, heat-inactivated NHS.

**Figure 7:**
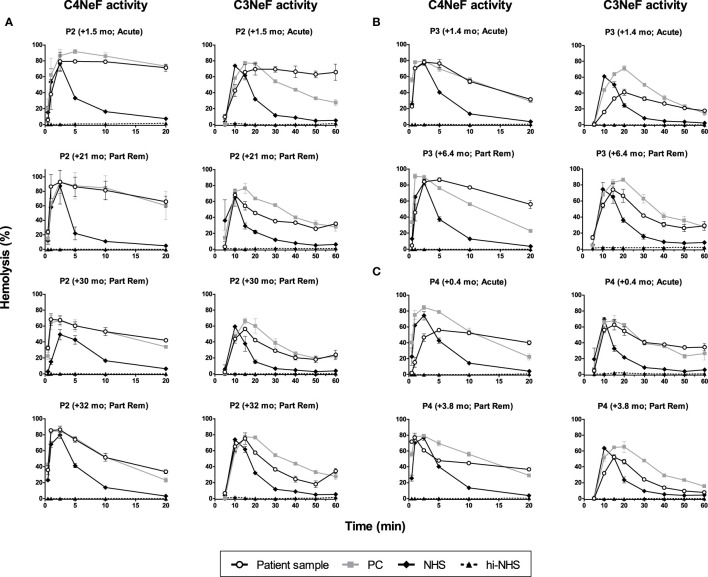
C4 nephritic factor and C3 nephritic factor activity of patients P2, P3, and P4 during follow-up. Samples from P2 **(A)**, P3 **(B)**, and P4 **(C)** collected over the disease course were tested for prolonged classical pathway activity, i.e. C4 nephritic factor (C4NeF) activity, and for prolonged alternative pathway activity, i.e. C3 nephritic factor (C3NeF) activity. Collection dates are presented as time after acute presentation in months (+mo). Hemolysis levels are presented as percentage of full lysis of erythrocytes in water. Results are shown as mean and range of two independent experiments. Part Rem, partial remission; PC, positive control; NHS, normal human serum; hi-NHS, heat-inactivated NHS.

We then monitored the activity of the AP and CP convertase-stabilizing factors during patient follow-up. Both the C4NeF and C3NeF activity of P1 (**Figure 6**), P2 (**Figure 7A**), P3 (**Figure 7B**), and P4 (**Figure 7C**) persisted over the disease course, up to 70 months of follow-up. Patients received standard therapy, including corticosteroids, mycophenolate mofetil, angiotensin converting enzyme (ACE) inhibitors, and diuretics, but no B cell-directed therapy or complement blockers such as eculizumab.

### Complement Activation Profiles During Patient Follow-Up

**Figure 8:**
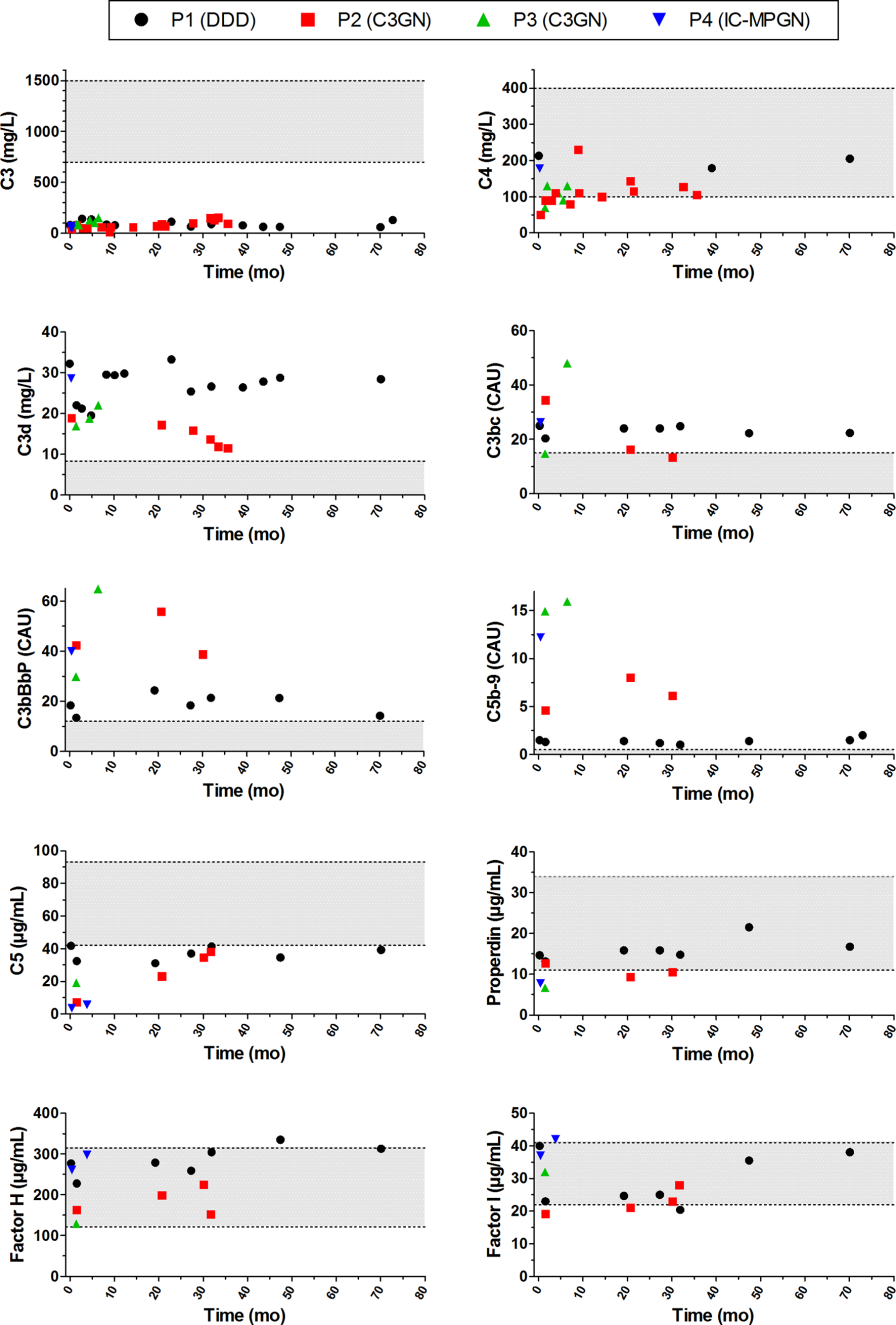
Complement activation profiles of the patients with prolonged classical pathway convertase activity during follow-up. Follow-up is indicated as time after initial presentation in months (mo). Grey areas indicate the healthy control ranges. DDD, dense deposit disease; C3GN, C3 glomerulonephritis; IC-MPGN, immune complex-mediated membranoproliferative glomerulonephritis; CAU, complement arbitrary units.

We also looked in detail at the complement profiles of the patients during follow-up by measuring the levels of the complement components, complement activation markers, and complement regulators. All four patients showed evident and consistent signs of complement activation, as indicated in the serum or plasma by very low C3 levels, elevated levels of the C3 breakdown products C3d and/or C3bc, elevated levels of C3bBbP (properdin-stabilized AP C3 convertase) and C5b-9, and decreased levels of C5 (**Figure 8**). In contrast to the persistent consumption of C3 in all patients analyzed, C4 levels were only decreased at presentation in P2 and P3. Properdin levels were decreased in P3 and P4 and in some samples of P2. The negative complement regulatory proteins Factor H and Factor I were generally in the normal range or slightly outside this range.

Compared to patients without C4NeF activity, the complement markers C3bBbP, C3bc, C5b-9, C5, and properdin tended to be more strongly increased or decreased in the four patients with C4NeF activity, especially when compared to patients without C3NeF activity (**Supplementary Figure 2**). When comparing the patients with both C4NeF and C3NeF activity with patients with C3NeF activity alone, the low C5 and properdin levels were most noticeable.

### Clinical Features During Follow-Up

#### Patient 1

This male patient presented at the age of 7 years with macroscopic hematuria and nephrotic range proteinuria with normal renal function. C3 serum levels were low (60 mg/L) but with normal serum C4 (260 mg/L). Serology findings for antineutrophil cytoplasmic antibody (ANCA), antinuclear antibody (ANA), and antistreptolysin O (ASO) titer were all negative. The kidney biopsy findings were consistent with DDD. With treatment including intravenous methylprednisolone therapy for 3 days followed by oral steroids, ACE inhibitor, and hydrochlorothiazide he reached partial remission, i.e. normal renal function with still some mild persisting proteinuria. His follow-up described in this study, however, started at the age of 15 years, when he presented again with nephrotic range proteinuria. This was preceded by diarrhea followed by general fatigue 5 weeks before presentation. C3 serum levels were again low (82 mg/L), C3d levels elevated (32.2 mg/L), and serum C4 levels normal (213 mg/L). This time exanthemata-like, non-itchy skin lesions were present at his face, trunk, arms, and legs as well as a staggering painful knee and ankles without redness and swollenness. Skin biopsy showed neutrophilic dermatosis. Fever developed during intravenous methylprednisolone treatment. Blood culture demonstrated a *N. meningitidis* C infection for which he was treated with antibiotics. No drusen were found with ophthalmologic examination. Intravenous methylprednisolone was given and followed by oral steroids, mycophenolate mofetil, and ACE inhibition. The urinary protein/creatinine ratio declined from 2000 mg/mmol in the acute phase to 486 mg/mmol after 5 months and 65 mg/mmol after 1 year. His serum albumin rose from 15 g/L in the acute phase towards 38 g/L 8 months after starting his treatment for the relapse. His renal function remained normal during this period with serum creatinine levels between 36-45 µmol/L. Low complement C3 levels as well as increased C3d levels persisted despite improvement in proteinuria. At the age of 18 years the patient was transferred to the adult nephrology clinic with normal serum creatinine (40 µmol/L), a urinary protein/creatinine ratio that varied between 69-132 mg/mmol, and normal blood pressure while receiving mycophenolate mofetil and ACE inhibition.

#### Patient 2

This girl was diagnosed at the age of 16 years with nephrotic syndrome with normal estimated glomerular filtration rate (eGFR) and was treated with a high dose of steroids and mycophenolate mofetil. Kidney biopsy taken on admission showed a mesangial proliferative glomerulonephritis with some global glomerulosclerosis and subepithelial humps. No staining for IgA, IgG, IgM, kappa or lambda light chains on immunofluorescence, marginal staining in capillaries for C1q and C3, but strong staining for C4d along the glomerular basement membrane were observed. Diffuse intramembranous, electron dense deposits were present on electron microscopy. On admission low serum C3 (40 mg/L), low C4 (50 mg/L), high C3d (18.9 mg/L) as well as low AP50 and CH50 activity were observed, and these values did not change during her three year follow-up, except for C4 normalization within one year. Although in follow-up she did not have peripheral and general edema, her serum albumin remained low (21-27 g/L) and her urinary protein/creatinine ratio ranged between 160-450 mg/mmol. Another renal biopsy performed at the age of 18 years demonstrated the pattern of C3GN with still extensive mesangial proliferation with mild endocapillary hypercellularity as well as endothelial, epithelial, intramembranous, endothelial, and mesangial depositions. Immunofluorescence showed this time C3 3+ next to C1q 1+, IgG, and IgM 1+. Unfortunately, no staining for C4d was performed. No glomerulosclerosis or interstitial fibrosis or tubular atrophy were seen. While on low dose of steroids, mycophenolate mofetil, losartan, and hydrochlorothiazide, she was transferred at 18 years to the adult nephrology clinic having a serum creatinine of 80 µmol/L and being normotensive but still with nephrotic range proteinuria.

#### Patient 3

This female patient presented at the age of 10 years at the pediatric nephrology clinic. She had periorbital edema for already 2-3 weeks. At presentation she had a normal renal function with an eGFR of 136 ml/min/1.73 m^2^ (serum creatinine 41 µmol/L) with nephrotic range proteinuria (urinary protein/creatinine ratio 1656 mg/mmol) and microscopic hematuria. She started with oral steroids for the presumed minimal change nephrotic syndrome. After 4 weeks of treatment she had responded partially with decline in proteinuria (urinary protein/creatinine ratio 717 mg/mmol) and no peripheral edema. Serum complement C3 level and C4 were very low (90 mg/L) and low (70 mg/L), respectively, and serum C3d level was elevated (16.9 mg/L). Serology findings for ANCA, ANA, and ASO titer were all negative. Kidney biopsy demonstrated a mesangioproliferative glomerulonephritis with no chronic lesions. C3 was prominently present on immunofluorescence microscopy (3+), and electron microscopy showed no intramembranous dense deposits. The referring center diagnosed the patient as C3GN. Mycophenolate mofetil was added to the steroids therapy followed by ACE inhibition, and later losartan was added. Six months later she is feeling well with no edema and a normal renal function, normotensive but still nephrotic range proteinuria (urinary protein/creatinine ratio 200-400 mg/mmol). The serum C3 level (150 mg/L) and C4 level 130 mg/L) are still low, and C3d remained elevated (22 mg/L). Her treatment consists of daily low steroids, mycophenolate mofetil, ramipril, and losartan.

#### Patient 4

This boy presented at the age of 15 years with nephrotic range proteinuria, microscopic hematuria, and general edema at the emergency department. Blood pressure was 126/86 mmHg. One week before admission he had a possible throat infection for which he visited the general practitioner. His medical history mentioned an autism spectrum disorder and mental retardation. His laboratory results showed a low serum albumin (14 g/L) and low-normal eGFR 81 ml/min/1.73 m^2^. The urinary protein/creatinine ratio on admission was 760 mg/mmol. Serology findings for ANCA, ANA, and ASO titer were all negative. Serum C3 was low (68 g/L), C3d elevated (28.6 mg/L), and C4 normal (178 mg/L). Due to the extreme fluid overload (estimated 18 L), fluid and salt restriction as well as diuretics were immediately started. Differential diagnosis mentioned post-infectious glomerulonephritis, IgA nephropathy, lupus nephritis, and C3G. Several attempts to perform a kidney biopsy under anesthesia in the acute phase of the disease failed due to his persistent refusals. In the light of the negative results for autoimmune diseases (see above) in combination with very low serological C3 level, elevated complement activation markers, and the presence of C3NeF, C3G was suggested. Treatment including high dose of steroids, mycophenolate mofetil, ACE inhibition, and diuretics was started. Fortunately, three weeks after the start of therapy a renal biopsy could be performed and showed an IC-MPGN without extracapillary proliferations and no interstitial-tubular damage. Immunofluorescence demonstrated granular deposits in the capillary wall: C3 3+, IgG 2+, IgM 1/2+, lambda 2+, kappa 1/2+, with no IgA or C1q. No chronic lesions were observed. Electron microscopy showed subendothelial electron dense deposits and presence of neutrophils. While on mycophenolate mofetil and ACE inhibition, three months later his serum albumin showed an increase (22 g/L) and his urinary protein/creatinine ratio (610 mg/mmol) some decrease.

## Discussion

The C3G classification was introduced to replace the older term MPGN in order to increase awareness of the AP dysregulation as the central event in disease pathogenesis. Most frequently, AP-directed autoantibodies are recognized as the underlying cause. In most cases C3NeFs are identified, but in fewer cases autoantibodies directed against Factor H, Factor B, or C3b can be detected (2, 33). C3G is a very heterogenous disease and is closely related to IC-MPGN. Although the autoimmune factors causing AP dysregulation have been widely studied, less research has focused on CP-specific dysregulating factors. Therefore, we aimed to explore the involvement and characteristics of factors dysregulating the CP convertase in C3G and IC-MPGN.

We validated a reliable and time-effective approach to screen for CP convertase-stabilizing factors in the samples of 52 patients whose samples were sent for C3G/IC-MPGN workup. We found four patients whose sera showed prolonged CP convertase activity: three patients with C3G and one patient with IC-MPGN. In three patients (P1, P2, and P4), we could confirm that Igs, i.e. autoantibodies named C4NeFs, were responsible for prolonged CP convertase activity, since the Igs from these patients induced convertase overactivation in NHS. This low prevalence of C4NeF of 6% is in line with reported data in other C3G/(IC-)MPGN patient cohorts, where the C4NeF prevalence was 3-19% (16, 17, 19, 34). However, the Igs from one of our patients (P3) did not support convertase stabilization when added to NHS. This indicates that serum factors other than Igs were present and responsible for the convertase overactivation in this patient. Future research should aim at identifying the exact nature of these types of serum factors. We hypothesize that this patient may have a genetic variation in CP convertase proteins or CP regulatory proteins, as has been described for a gain-of-function mutation in C2 (35). Of note, we did not find any aberrations in the *C2* gene of patient P3.

Interestingly, all four patients showing prolonged CP convertase activity also showed prolonged AP convertase activity caused by C3NeFs. Such double positivity has been shown previously in 20% (17), 41% (16), and 53% (19) of C4NeF-postive C3G/MPGN patients, although isolated C4NeF has also been reported in these studies. This overlap raises the question of whether or not C3NeF and C4NeF activities can be caused by the same antibody, at least in some cases. Using C3-depleted serum, we observed that the Igs of our C4NeF-positive patients (P1, P2, and P4) could stabilize the C4bC2a CP convertases. These convertases do not contain components (epitopes) that are present in the C3bBb(P) convertases of the AP, which are stabilized by C3NeFs. Furthermore, in previous studies we have convincingly demonstrated that this C4NeF detection assay specifically measures CP and not AP activity. Convertase formation in this assay is completely abolished by depletion of the CP components C1q and C2 in test serum, while depletion of the AP component Factor B has no significant effect (15, 35). Similarly, the C3NeF positivity of patient Ig samples was determined in an AP-specific assay with a buffer containing magnesium but lacking calcium. This eliminates CP activity and only allows formation of AP convertases, which do not contain C4NeF epitopes. Thus, these data strongly suggest that the C3NeF and C4NeF activity in these patients is caused by different clones of autoantibodies, rather than by an autoantibody from a single B-cell clone.

Next to C3NeF and C4NeF activity, P2 and P4 also had rare heterozygous genetic aberrations, which may indicate that multiple factors have precipitated disease in these patients. Genetic analysis revealed a missense variant in *C3* [c.691A>C (p.Ser231Arg)] in patient P2, which has not been described previously and is predicted as probably pathogenic by mutation prediction software (SIFT, Align, and PolyPhen). In patient P4, a frameshift variant in *CFHR5* [c.485_486dup (p.Glu163Lysfs*10)] was found, which leads to a truncated protein. Similar frameshifts (p.Glu163Argfs*35) have been previously described in C3G patients (36, 37) and in one patient with atypical hemolytic uremic syndrome (38).

Overall, we observed that the patients with C4NeF activity consistently showed strongly aberrant convertase activity and complement biomarker profiles. First, in all four patients the C3NeF and C4NeF activity remained present during the follow-up, even though the patients all reached partial remission under therapy. Of note, the C4NeF-positive patients we described did not show an abnormal clinical presentation compared to C3G/IC-MPGN patients without C4NeFs. Second, the complement biomarker profiles during follow-up revealed strong, persistent complement AP activation in all patients. C3 was highly consumed and also the C5 levels were below control range, whereas the activation markers C3bc, C3d, C3bBbP, and C5b-9 were all elevated. This indicates that complement was activated up to the level of the terminal pathway and is consistent with previous reports of C4NeF-positive C3G/IC-MPGN patients (16, 17, 34). Previously, Zhang et al. showed that in their C3G cohort all the C4NeF-positive patients showed low properdin levels (17). This was also the case for the C4NeF-positive C3G patient found by Blom et al. (34). In our study, three patients showed properdin levels that were below the cut-off values. A highly activated complement profile, as seen here in the C4NeF-positive patients, can also be observed in certain C4NeF-negative (often C3NeF-positive) C3G/IC-MPGN patients, but not in all and not always equally strong. Prospective studies with longitudinal data collection are required to further understand the role of C4NeF in C3G and IC-MPGN pathogenesis and to investigate if C4NeF activity correlates with clinical parameters and complement profiles.

Although AP activation persisted throughout follow-up in all four patients, C4 levels were only decreased in two patients at onset of the disease but normal in most of the other samples analyzed. Normal C4 levels in patients with C4NeF have frequently been reported before, and this is not surprising, since both C3NeF and C4NeF stabilize C3 convertases and eventually drive AP activation (16, 17, 34). Mechanisms known to promote C4 consumption such as infection may play a role at the beginning of the disease but are not continuously present in the patients, whereas intrinsic C3 activation drivers are (C3NeFs, C4NeFs, and in some cases genetic changes).

The main advantage of the assay for detection of CP convertase-dysregulating factors described here is that the activity of the CP convertases is examined in conditions in which all serum factors are present to interact with the convertases. This allowed us to be the first to identify factors prolonging CP convertase activity that were not Igs in P3. Interestingly, we also found another patient with non-Ig-mediated CP convertase stabilization in the group of patients with unknown diagnosis. This patient did not show C3NeF activity and thus had isolated CP convertase dysregulation. Unfortunately, no clinical data could be retrieved from this patient. Nonetheless, it underlines that such factors would have been missed if only Ig fractions from patients were tested. Furthermore, C4NeFs form a heterogenous group, as do the C3NeFs: C4NeFs can prevent the intrinsic and extrinsic decay of convertases to different extents by interfering with different complement regulators (17, 24, 34, 39–41). Therefore, studying the physiological environment of complete serum enhances the likelihood of detecting relevant factors. Finally, this serum approach allows a quick result and requires only a very small amount of patient material.

In this study we have demonstrated that in C3G and IC-MPGN CP convertase dysregulation may occur. Screening for these abnormalities should be performed to carefully characterize the complement defect in each patient which may affect the choice of treatment. What still remains unknown is why and how individual patients develop C4NeFs and to what extent they contribute to the disease course, also when other factors such as C3NeFs are present. C3G is a typical AP-mediated disease, but it is often preceded by an infectious trigger. It is possible that the presence of C4NeFs (or non-Ig serum factors with C4NeF-like activity) exaggerate the initial (weak) CP-mediated response, after which the disease evolves to a primarily AP-driven disease process *via* the AP amplification loop, especially if also C3NeFs are present. Similarly, it has been described that some patients with post-infectious glomerulonephritis may evolve to C3G (42, 43).

In conclusion, we have shown in-depth analysis of CP convertase activity in our C3G/IC-MPGN patient cohort and found that CP convertase overactivation can be caused by C4NeFs but also by serum factors other than Igs that are still to be identified. Patients with prolonged CP convertase activity additionally showed AP dysregulation as seen by C3NeF activity and clear signs of full complement activation in their blood. We recommend that all patients with C3G and related diseases such as IC-MPGN are tested for CP convertase-stabilizing factors for full characterization of the underlying complement defect, especially in the light of a broad spectrum of upcoming complement-inhibitors targeting the complement pathways at different levels (44). Furthermore, future research is needed to elucidate the exact role of CP convertase-stabilizing factors in the initiation or progression of disease.

## Data Availability Statement

The original contributions presented in the study are included in the article/**Supplementary Material**. Further inquiries can be directed to the corresponding author.

## Ethics Statement

The studies involving human participants were reviewed and approved by ethics committee of the Radboud University Medical Center.

## Author Contributions

MM: Methodology, formal analysis, investigation, writing – original draft, and visualization. NK: Conceptualization, resources, and supervision. SK: Investigation. SS: Investigation. VG: Resources. FE: Resources. ED: Resources. HD: Resources. CD: Resources. JW: Resources and writing – review and editing. BH: Conceptualization, project administration, supervision, and writing – review and editing; EV: Conceptualization, supervision, methodology, and writing – review and editing. All authors contributed to the article and approved the submitted version.

## Funding

This work was supported by the Dutch Kidney Foundation (13OCA27 COMBAT Consortium).

## Conflict of Interest

JW has received grants from Achillion and Chemocentryx, outside the submitted work.

The remaining authors declare that the research was conducted in the absence of any commercial or financial relationships that could be construed as a potential conflict of interest.

## Publisher’s Note

All claims expressed in this article are solely those of the authors and do not necessarily represent those of their affiliated organizations, or those of the publisher, the editors and the reviewers. Any product that may be evaluated in this article, or claim that may be made by its manufacturer, is not guaranteed or endorsed by the publisher.
