# Supplementary material for: Different Aspects of Classical Pathway Overactivation in Patients With C3 Glomerulopathy and Immune Complex-Mediated Membranoproliferative Glomerulonephritis
Source: Front Immunol. 2021 Aug 11;12:715704. doi: 10.3389/fimmu.2021.715704 (PMC8386118; doi:10.3389/fimmu.2021.715704)
Supplement: Supplementary file 1 [file DataSheet_1.docx]

Supplementary Material

##
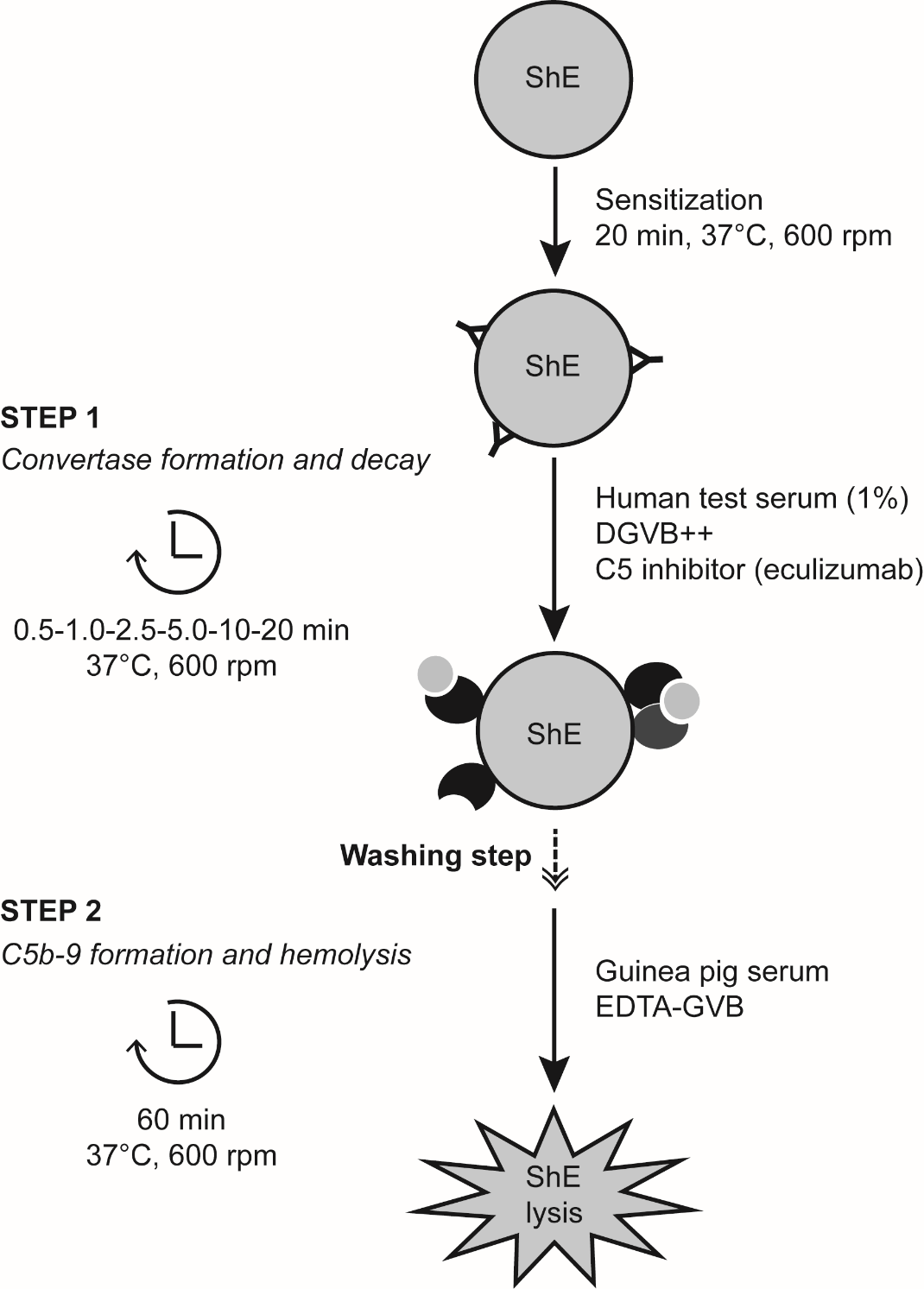


**Supplementary Figure 1.** Schematic overview of the classical pathway convertase activity assay using sheep erythrocytes (ShE). DGVB++, dextrose gelatin veronal buffer; EDTA-GVB, ethylenediaminetetraacetic acid–gelatin veronal buffer.

**
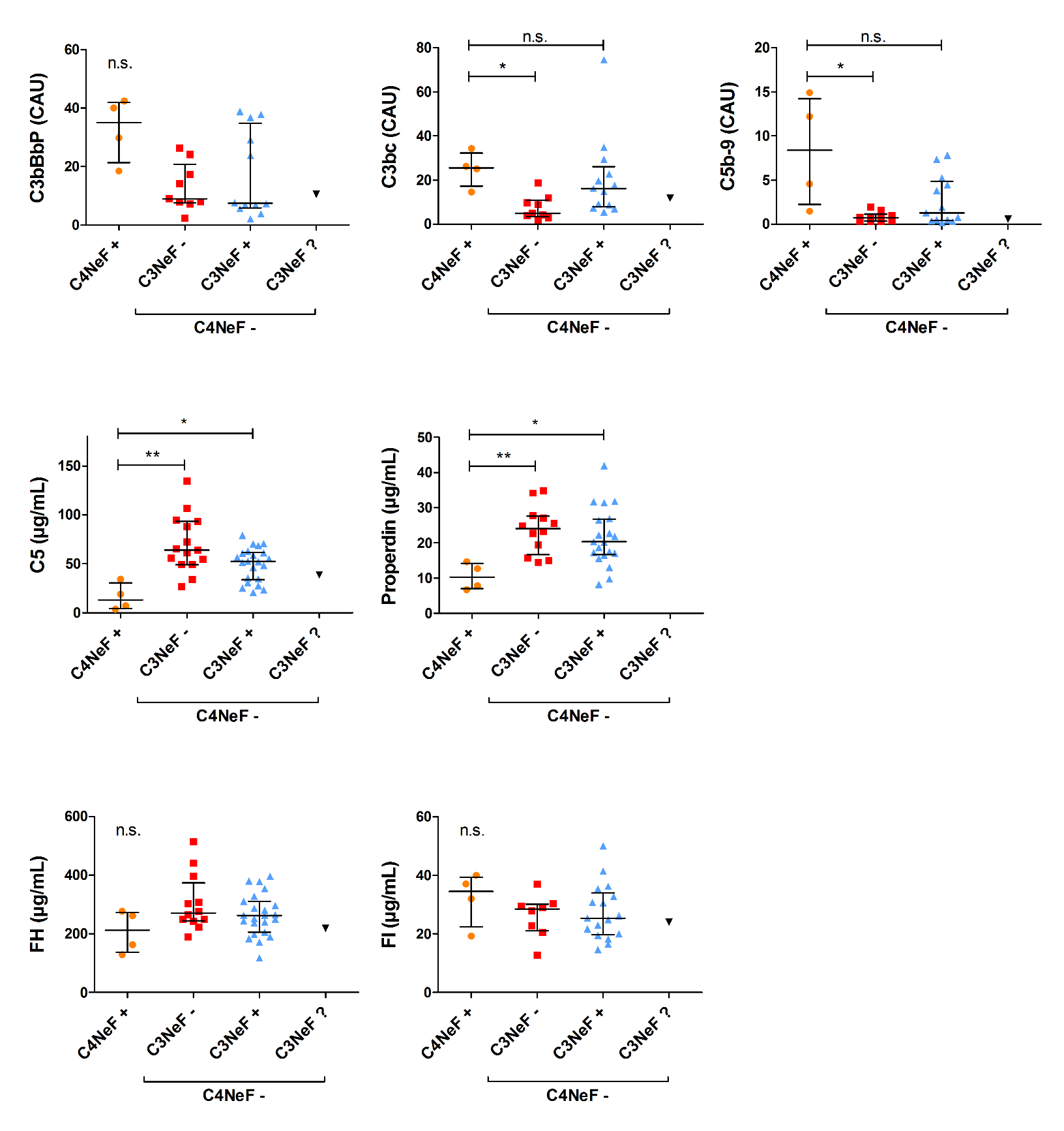
**

**Supplementary Figure 2.** Comparison of complement marker profiles in the C3 glomerulopathy/immune complex-mediated membranoproliferative glomerulonephritis cohort. The levels of the complement activation markers C3bBbP, C3bc, and C5b-9, complement component C5, positive regulator properdin, and negative regulators Factor H (FH) and Factor I (FI) were compared between patients with prolonged classical pathway convertase activity, i.e. C4 nephritic factor activity (C4NeF +), and patients without C4NeF activity (C4NeF - ) and with or without C3 nephritic factor activity (C3NeF + or C3NeF -, respectively). Note that the C4NeF + patients also were C3NeF +. The data shown are from the time of C4NeF investigation. Error bars represent the median and interquartile range. Statistical differences were calculated using the Kruskal-Wallis test (excluding the C3NeF unknown (C3NeF ?) group), and if significant Dunn’s post-test was performed: n.s., not significant; *, P<0.05; **, P<0.01. CAU, complement arbitrary units.
